# Assessment of Fetal Dose and Health Effect to the Fetus from Breast Cancer Radiotherapy during Pregnancy

**DOI:** 10.3390/life12010084

**Published:** 2022-01-07

**Authors:** Pattarakan Suwanbut, Thiansin Liamsuwan, Danupon Nantajit, Wilai Masa-nga, Chirapha Tannanonta

**Affiliations:** 1 Princess Srisavangavadhana College of Medicine, Chulabhorn Royal Academy, Bangkok 10210, Thailand; pattarakan.suw@cra.ac.th (P.S.); danupon.nan@cra.ac.th (D.N.); 2 Radiation Oncology Department, Chulabhorn Hospital, Chulabhorn Royal Academy, Bangkok 10210, Thailand; wilai.mas@pccms.ac.th (W.M.-n.); chirapha.tan@pccms.ac.th (C.T.)

**Keywords:** fetal dose, Monte Carlo simulation, computational phantom, deterministic effect, stochastic effect

## Abstract

Decision for radiotherapy during the first trimester of pregnancy may occur, as patients may not realize their pregnancy at the very early stage. Since radiation dose can affect fetal development, the aim of this study was to evaluate fetal dose and associated deterministic effects and risks to the fetus from breast cancer radiotherapy of an 8-week pregnant patient. PHITS (Particle and Heavy Ion Transport code System) Monte Carlo simulation and the J-45 computational pregnancy phantom were used to simulate breast cancer radiotherapy from a 6 MV TrueBeam linear accelerator using the three dimensional-conformal radiotherapy (3D-CRT) technique with a prescribed dose to the planning target volume (PTV) of 50 Gy. Once the fetal dose was evaluated, the occurrence of the deterministic effects and risks for developing stochastic effects in the fetus were assessed using the recommendations of NCRP Report No. 174, AAPM Report No. 50, and ICRP Publication 84. The fetal dose was evaluated to be 3.37 ± 2.66 mGy, suggesting that the fetus was expected to have no additional deterministic effects, while the risks for developing cancer and malfunctions were similar to that expected from exposure to background radiation. The comparison with the other studies showed that accurate consideration of fetal position and size was important for dose determination in the fetus, especially at the early pregnancy stage when the fetus is very small.

## 1. Introduction

In general, radiotherapy treatment planning for cancer patients considers the in-field radiation dose, including a high dose to the treatment volume for high tumor control and a low dose to normal tissues for reducing tissue complications in order to achieve a high therapeutic ratio. However, sometimes pregnant patients may receive radiotherapy, causing additional concern for out-of-field radiation dose outside the treatment volume, because this radiation dose will also affect the fetus. Therefore, the treatment plan must consider both the in-field radiation dose for the patient and the out-of-field radiation dose in order to avoid deterministic effects and minimize the risk of developing stochastic effects in the fetus.

According to the American Association of Physicists in Medicine Task group 36 (AAPM TG-36), most pregnant cancer patients are diagnosed with breast cancer, melanoma, lymphoma, leukemia, uterine cancer, thyroid cancer, and brain cancer [1]. During radiotherapy treatment, the fetus should not receive a dose exceeding 100 mGy, which is the threshold dose for deterministic effects. Increased fetal dose also translates to more likelihood that the fetus will develop cancer [1]. 

The in-field radiation dose is subjected to evaluation in the treatment planning process, while there are some difficulties evaluating the out-of-field fetal dose. First, in-vivo measurements of the fetal dose only provide the point dose instead of the dose in the fetal volume. However, risk assessment should be done from the organ dose rather than the point dose [2]. Moreover, in-vivo measurements are invasive for patients and are only possible for patients in the first trimester of pregnancy [3]. Second, although phantom measurements of fetal dose are usually performed [4], these measurements only give the point dose and the measured fetal dose could be unrealistic because phantoms are made of tissue-equivalent materials. Furthermore, if detectors are not specifically calibrated for the out-of-field dose, uncertainties in dose measurement are likely to occur [1,4].

The out-of-field dose cannot be estimated using treatment planning systems. A comparison of measured doses with those calculated by a treatment planning system showed that the difference of dose could exceed 30% even at 3 cm distance from the field edge, and the discrepancy increases with increasing distance from the field edge [5].

To overcome the aforementioned limitations, Monte Carlo simulations with actual patient information and radiation field characteristics can help to achieve an accurate assessment of the fetal dose. 

The objective of this study was to assess the fetal dose and the associated deterministic effects and risks from breast cancer three-dimensional conformal radiotherapy (3D-CRT) at 8 weeks post conception using PHITS (Particle and Heavy Ion Transport code System) Monte Carlo simulation code version 3.24 and the J-45 computational pregnant female phantom [6]. The simulated treatment head was a 6 MV Varian TrueBeam linear accelerator (Varian Medical System. Inc., Palo Alto, CA, USA). The gestation stage of 8 weeks post conception was selected because it is a period when the fetus is highly sensitive to radiation and, in many cases, patients do not show any physical characteristics of pregnancy. Therefore, these radiotherapy patients are likely to receive treatment without consideration of the fetal dose. 

## 2. Materials and Methods

### 2.1. Treatment Planning

**Figure 1:**
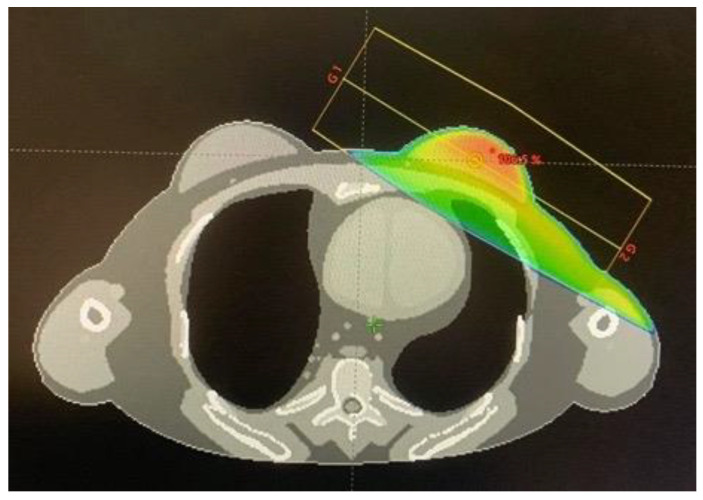
The dose distribution of the treatment plan calculated by the Eclipse treatment planning system.

Eclipse treatment planning system version 16.0 (Varian Medical System. Inc., Palo Alto, CA, USA) was used for creating the treatment plan. First, the J-45 pregnant female phantom at 8 weeks post conception [6] was converted to a DICOM-CT dataset using an in-house developed MATLAB program (The MathWorks, Inc., Natick, MA, USA). The converted CT dataset had the dimension of 47.124 cm × 26.712 cm × 152.55 cm with a voxel size of 0.126 cm × 0.126 cm × 0.27 cm. The mid-line point of the fetus was 32.5 cm from the central axis or 25 cm from the field border. Treatment planning was done by importing the CT dataset of the J-45 pregnant female phantom to the treatment planning system. The target and organs at risk for treatment planning were contoured, including the left breast (whole breast), which was considered as the planning treatment volume (PTV), as well as the heart, left lung, right lung, total lung, spinal cord, and esophagus as the organs at risk. The treatment plan was calculated for 6 MV 3D-CRT with the prescribed dose in the PTV of 50 Gy (2 Gy/fraction × 25 fraction), the dose rate of 600 MU/min, the gantry rotation angles of 119.9° and 299.9° (tangential fields) at 100 cm source to axis distance (SAD), and the field size of 9 × 15 cm^2^. The dose-volume criteria for the target and organs at risk were set following QUANTEC guidelines [7], as shown in Table 1. Figure 1 shows the treatment plan obtained in this work.

### 2.2. Monte Carlo Simulation

Monte Carlo simulations have been used for determination of the out-of-field dose for various treatment techniques and linear accelerator models [5]. In this work, PHITS version 3.24 [8] was used for the simulation. The simulation used the phase space files above the movable upper jaws of the 6 MV TrueBeam linear accelerator as the radiation sources, while the treatment head components downstream of the phase space surface were modelled in detail according to the manufacturer’s blueprint [9]. The validation of the simulated treatment head was done by comparing a set of simulated results with experimental data. For in-field dose calculations, the calculated percentage depth dose distributions and the lateral dose distributions were validated. In addition, for the out-of-field dose calculations, AAPM TG-158 recommends that the Monte Carlo simulation should also be validated in terms of the dose near the phantom surface and the peripheral dose. The simulated treatment head used in this work has been successfully validated for both in-field and out-of-field dose, as reported previously [10].

**Figure 2:**
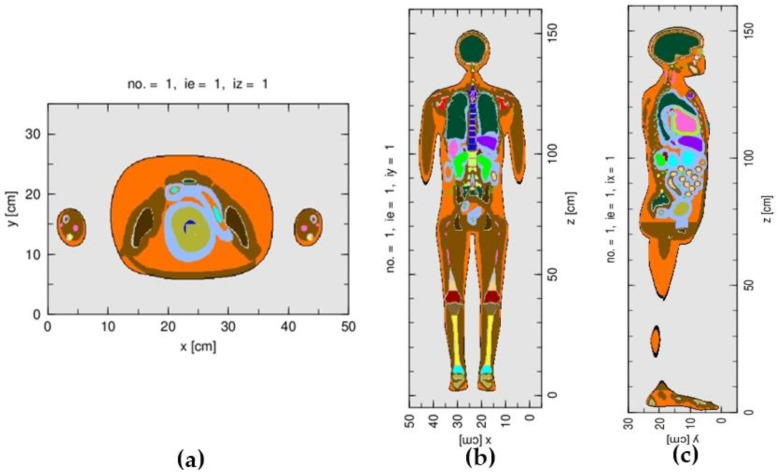
The graphical outputs of the J-45 pregnant female phantom at 8 weeks post conception: (**a**) axial plane, (**b**) coronal plane, and (**c**) sagittal plane.

For the calculation of the fetal dose, the J-45 pregnant female phantom at 8 weeks post conception was integrated into the treatment head simulation, by positioning the phantom relative to the treatment head, as described by the treatment plan. In the Monte Carlo simulation, the phantom, instead of the treatment head, was rotated to represent the gantry rotations of 119.9° and 299.9°. The simulated number of source particles at the phase space surface was in the range of 4.8 × 10^7^ in order to produce reasonable statistical uncertainties of the simulated results for most organs. The number of simulated particles was limited by the number of converted sources given in the phase space file, or otherwise the result would have been biased. Figure 2 shows the graphical output of the simulated J-45 phantom. 

The fetal dose and dose in the target and organs at risk of the patient were calculated in the structures of interest. Each organ of the J-45 phantom was defined as a “universe” in PHITS. A combination of relevant organs (or universes) was carried out to define a structure of interest for the dose calculation, as listed in Table 2. The statistical uncertainties of the Monte Carlo simulated results were less than 0.39% for the target and 7.71% for the organs at risk. The calculated doses were normalized so that the mean target dose obtained from the Monte Carlo simulation and the treatment plan were equivalent.

### 2.3. Evaluation of Deterministic Effect and Stochastic Effect

Once the fetal dose was evaluated from the simulation, the occurrence of deterministic effects and the risk for developing stochastic effects were assessed using the recommendations of NCRP Report No. 174 [11], AAPM TG-36 [1], and ICRP Publication 84 [12], as described in Table 3.

## 3. Results

### 3.1. Determination of Fetal Dose

Table 2 shows the doses and volumes of the target and organs at risk obtained from the treatment plan described in Section 2.1 and the Monte Carlo simulation. The organ volumes from both calculations agreed within 4.8%. Small deviations arose from manual contouring during the treatment planning process. From Table 2, doses in the organs at risk from the treatment plan and the Monte Carlo simulation passed the criteria specified in Table 1. The differences of doses in the organs at risk obtained from both calculations ranged from 6.38–92.31%, increasing with the increasing distance from the target. From this treatment plan, the simulated absorbed dose in the fetus was found to be 3.37 mGy ± 2.66 mGy. For comparison, the calculated doses in the uterus, urinary bladder, and small intestine, which were located close to the fetus, but were larger in size than the fetus, are also given in Table 2. The absorbed doses in the latter organs were 1–2 orders of magnitude larger than the fetal dose.

### 3.2. Deterministic Effects and Stochastic Effects of the Fetus

The occurrence of deterministic effects and the risks for developing stochastic effects were assessed using the recommendations of NCRP Report No. 174 [11], AAPM TG-36 [1], and ICRP Publication 84 [12] (Table 3). The calculated fetal dose of 3.37 ± 2.66 mGy was much lower than the threshold dose for the occurrence of deterministic effects of 100 mGy, as recommended by NCRP Report No. 174 [11]. Therefore, no further deterministic effects from the baseline level were expected due to this radiation exposure. According to ICRP Publication 84 [12], the probability that a child will have no malformation and the probability that a child will not develop cancer would be 97% and 99.7%, respectively, similar to the effects of exposure to a background radiation dose. Lastly, according to AAPM TG-36 [1], with the calculated fetal dose of less than 5 cGy, there was little risk of damage to the fetus.

## 4. Discussion

### 4.1. Determination of Fetal Dose

#### 4.1.1. Comparison of the Monte Carlo Simulation and the Treatment Plan

The differences of doses in the organs at risk obtained by the Monte Carlo simulation and the treatment planning system were in the range of 6.38–92.31% for the left lung, total lung, esophagus, heart, right lung, and spinal cord, respectively (Table 2). The larger dose differences were observed for the organs at further distances from the field edge. Such dose differences between Monte Carlo simulations and treatment planning systems have been reported in the literature in relatively good agreement with this work, e.g., the dose differences in the organs at risk have been reported to range from 10–70% for the heart, right lung, esophagus, and left lung, respectively, for a breast cancer treatment plan [12]. Moreover, the difference of dose in the heart was found to be relatively large (22.07%), because it was the penumbra region, as shown in Figure 1, where the dose from the treatment plan could be underestimated [13].

The differences in the mean doses in the out-of-field organs obtained by the Monte Carlo simulation and the treatment planning system may arise because of several reasons. First, treatment planning systems are known to underestimate the collimator scatter and scatter from other components of the beam line, which directly affect the out-of-field dose calculations. Therefore, for organs further away from the target, the dose discrepancies increased, in accordance with the previous studies [14,15]. Second, in the treatment planning process, manual contouring was done to delineate the organs and structures of interest. This step may cause errors resulting in differences in the organ volumes, as shown in Table 2. Nevertheless, the volume difference was at a maximum of 4.8%. Therefore, the manual contouring only minimally contributed to the dose discrepancies. Lastly, in this study, Monte Carlo simulation was validated for a 0° gantry angle but the treatment plan was calculated using tangential fields. A previous study [16] showed that oblique beams or tangential fields may cause deviations between the doses obtained from the Monte Carlo simulations and the treatment planning systems if using a half phantom. However, deviations between both calculation techniques were insignificant if using a full phantom, which was the case for the treatment plan for the patient done in this study.

#### 4.1.2. Fetal Dose Evaluation Using the Monte Carlo Simulation

The simulated fetal dose based on the treatment plan with the J-45 pregnant female phantom at 8 weeks post conception was 3.37 ± 2.66 mGy, corresponding to 0.007% of the target dose. Several studies have attempted to determine the fetal dose obtained during radiotherapy. For example, Antypas et al. [3] reported a fetal dose of 3.91 cGy (0.085% of the target dose) from breast cancer radiotherapy at 2 weeks post conception using an in vivo measurement method with TLDs. In that measurement, treatment was done with 3D-CRT using 6 MV photons from SL75-5 linac and the prescribed dose of 46 Gy (2 Gy/fraction) using tangential fields, with gantry angles of 295° and 120° and a field size of 7 × 15 cm^2^. In another study, Mazonakis et al. [2] reported a fetal dose of 4.40 cGy (0.088% of the target dose) from 3D-CRT breast cancer radiotherapy at the first trimester (1–3 months) post conception using the MCNP Monte Carlo simulation code and a computational phantom. The simulation was done for 6 MV photons from SL75-5 linac and the prescribed dose of 50 Gy (2 Gy/fraction) using tangential fields with gantry angles of 285° and 110° and a field size of 10.5 × 19 cm^2^. 

Although the studies by Antypas et al. [3] and Mazonakis et al. [2] investigated the different stages of pregnancy, the percentage fetal doses relative to the target doses were relatively similar between both studies (0.085% and 0.088%, respectively), and much higher than those obtained in this study (0.007%). It is of note that the different gestation stage, linear accelerator model, and treatment plan were used in this study compared with the studies of Antypas et al. [3] and Mazonakis et al. [2]. Moreover, the reason for the relatively large difference in percentage fetal dose may lie in the fact that when using different generations of computational phantoms, such as the stylized phantom used by Mazonakis et al. [2] and the J-45 pregnant female phantom at 8 weeks post conception used in this study, the resulting dose could be different [17]. In addition, from the Monte Carlo simulation of Mazonakis et al. [2], it was likely that the fetal dose was estimated from the dose in the uterus, while it was not clear if the detector position used in the measurement of Antypas et al. [3] directly represented the fetus position. In many cases, the detectors were positioned at the fundus of uterus for the measurement of the fetal dose [3,17,18]. In that case, the calculation in this work also gave a uterus dose of 0.088% of the prescribed dose, similar to those reported by both studies. 

In this study, the statistical uncertainty of the calculated fetal dose was relatively large, namely, 78.9%. The increase of the source particles in the simulation did not significantly reduce the statistical uncertainties. Variance reduction techniques such as particle splitting could have been performed to improve the statistical uncertainty of the fetal dose. However, it is usually recommended to give an importance ratio between neighboring organs of less than 2–3. From the results shown in Table 2, the statistical uncertainty of the uterus dose was 7.7%, while the statistical uncertainty of the fetal dose was 78.9%. Moreover, the volume of the fetus was only 1.9% of the volume of the uterus. Therefore, to calculate a fetal dose with a lower statistical uncertainty, for example, less than 10%, similar to those for other organs, a large importance should be assigned to the uterus and neighboring organs in descending order relative to the fetus. However, this would increase the simulation time tremendously. 

The large statistical uncertainty of the fetal dose was because the distance of the fetal midline from the field border was relatively large, namely, 25 cm. Therefore, the fetus received a relatively low dose and, thus, the fetal dose had a relatively large statistical uncertainty. Moreover, the volume of the fetus at 8 weeks post conception was relatively small (3.2 cm^3^). Therefore, the probability that radiation would traverse the fetal volume should be relatively low. In comparison, for other out-of-field organs that were at similar positions as the fetus, namely, the small intestine, uterus, and urinary bladder, the statistical uncertainties of the calculated dose did not exceed 12.4%. In this case, the distances of the midlines of the small intestine, uterus, and urinary bladder from the field border were about 17, 23, and 35 cm, respectively. Although the out-of-field distances of the fetus and these organs were similar, there were large differences of the statistical uncertainties associated with the fetal and organ doses, which were due to the different sizes of these structures. As out-of-field organs were likely to receive a non-uniform dose, meaning that doses to tissue voxels in the organ volume were not necessarily similar, it was not unexpected that the mean doses to these organs and to the fetus, which had a relatively small volume, were largely different. Similarly, even though the fetus was in the uterus, the dose to the uterus was not necessarily uniform because it was positioned at a relatively large distance from the field edge. As a result, the mean dose to the fetus could be different from the mean dose to the uterus. In addition to the statistical uncertainty related to the Monte Carlo method, from the comparison of the simulated and measured peripheral dose in our previous study [10], it was found that both results at ±15 cm distances from the field edge were up to 33.33% different. In total, the uncertainty related to the calculated fetal dose was expected to be at least 85.67%.

In this work, the 3D whole breast radiotherapy was simulated because a computational phantom was used, making it difficult to define a realistic tumor volume. An IMRT plan could be investigated, which required the simulation of the movement of the multi-leaf collimators (MLC). In that case, the simulation with the MLC would need to be validated before use in the fetal dose evaluation. For future work, we plan to investigate the out-of-field dose and fetal dose received from the field-in-field (FIF) technique, also known as forward IMRT, which is the technique typically used for breast cancer treatment in our clinic. It should also be mentioned that advanced treatment techniques could produce a larger out-of-field dose than 3D-CRT [19].

### 4.2. Fetal Deterministic Effect and Stochastic Effect

From the Monte Carlo simulation, it was found that the fetus would receive a radiation dose from breast cancer radiotherapy with the prescribed 3D-CRT technique of 3.37 ± 2.66 mGy (0.007% of the prescribed dose), which was much lower than the threshold dose of 100 mGy for the occurrence of deterministic effects, and posed low risks for developing cancer and malformation. The result suggests that the treatment plan was appropriate as it gave the tumor treatment dose according to the prescription, with an expectedly very low complication probability of the fetus at 8 weeks post conception. If the pregnancy was at a later gestation stage, the fetus could have received a higher dose and lead shielding could be a choice for fetal dose reduction [20,21,22,23]. 

## 5. Conclusions

The absorbed dose in the fetus from breast cancer 3D-CRT of an 8-week pregnant patient was calculated for a 6 MV TrueBeam linear accelerator. The target was prescribed with a 50 Gy (2Gy/fraction) dose at SAD 100 cm using tangential fields, with gantry angles of 119.9° and 299.9° and the field size of 9 × 15 cm^2^. The fetal dose was calculated using PHITS Monte Carlo simulation and the J-45 pregnant female phantom at 8 weeks post conception. The simulation yielded a fetal dose of 3.37 ± 2.66 mGy, suggesting that, from this radiotherapy treatment plan, deterministic effects should not happen to the fetus and the fetus should have very low risks of 0.3% and 3% for developing cancer and malfunction, respectively. The Monte Carlo method for fetal dose determination as done in this work can be carried out for the fetus at other gestation stages, which will help clinicians and medical physicists to decide which treatment planning options and mitigation techniques to use to minimize the fetal dose. In fact, at a later gestation stage, the fetal volume increases and the evaluated fetal dose is expected to have lower statistical uncertainties compared to the result of this work. Future works should also consider fetal dose from advanced treatment techniques such as IMRT and VMAT, which could produce a larger out-of-field dose than 3D-CRT. In addition, the fetal dose obtained from different treatment modalities, such as particle therapy, can be studied. In that case, the out-of-field dose is contributed by both photons and neutrons.

## Figures and Tables

**Table 1:** Dose-volume criteria for the target and organs at risk.

Structures	Criteria
Target (Left breast, whole breast)	D_100_ = 95% prescribed dose
Left lung	D_Mean_ < 20 Gy
Right lung	D_Mean_ < 20 Gy
Total lung	D_Mean_ < 20 Gy
Heart	D_Mean_ < 6 Gy
Esophagus	D_Mean_ < 34 Gy
Spinal cord	D_Max_ < 45 Gy

**Table 2:** Volumes and doses in the structures of interest obtained from the treatment planning system (TPS) and the Monte Carlo simulation (MC). Percentage dose differences were calculated using the Monte Carlo simulation as the reference.

Structures	Volume in TPS (cm^3^)	Volume in MC (cm^3^)	Mean Dose in TPS (Gy)	Mean Dose in MC (Gy)	% Dose Difference
Target (Left breast, whole breast)	142.9	141.21	46.59	46.59 ± 0.18	-
Left lung	1172.8	1133.9	3.23	3.45 ± 0.02	−6.38
Total lung	2505.0	2530.0	1.49	1.69 ± 0.01	−11.83
Esophagus	32.8	31.3	0.21	0.27 ± 0.02	−22.22
Heart	540.7	543.6	1.66	2.13 ± 0.02	−22.07
Right lung	1446.2	1396.1	0.07	0.26 ± 0.01	−73.08
Spinal cord	40.2	41.5	0.01	0.13 ± 0.01	−92.31
Small intestine	-	976.2	-	0.11 ± 2.22 × 10^−3^	-
Uterus	-	169.3	-	0.04 ± 3.08 × 10^−3^	-
Fetus	-	3.2	-	3.37 × 10^−3^ ± 2.66 × 10^−3^	-
Urinary bladder	-	77.4	-	0.03 ± 3.72 × 10^−3^	-

**Table 3:** Deterministic effect and Stochastic effect related to the fetal dose obtained from NCRP report No. 174 [11], AAPM TG-36 [1], and ICRP Publication 84 [12].

NCRP Report No. 174 (6th to 13th Weeks)		
Radiation dose	Effect	
<0.10 Gy	Non-cancer health effects not detectable	
0.10–0.50 Gy	Growth restriction possible	
>0.50 Gy	Probability of miscarriage may increase, depending on dose Growth restriction is likely	
AAPM TG-36		
Radiation dose	Risk	
<0.05 Gy	Little risk of damage	
0.05–0.1 Gy	Risk uncertain	
0.1–0.5 Gy	Significant risk of damage	
>0.5 Gy	High risk of damage during all trimester	
ICRP Publication 84		
Absorbed dose to conceptus above natural background	Probability that the child will have no malformation	Probability that the child will not develop cancer^2^
(mGy)	(%)	(%)
0	97	99.7
0.5	97	99.7
1	97	99.7
2.5	97	99.7
5	97	99.7
10	97	99.6
50	97	99.4
100	97	99.1
