# Correction: Suwanbut et al. Assessment of Fetal Dose and Health Effect to the Fetus from Breast Cancer Radiotherapy during Pregnancy. *Life* 2022, *12*, 84

**DOI:** 10.3390/life13020285

**Published:** 2023-01-19

**Authors:** Pattarakan Suwanbut, Thiansin Liamsuwan, Danupon Nantajit, Wilai Masa-nga, Chirapha Tannanonta

**Affiliations:** 1 Princess Srisavangavadhana College of Medicine, Chulabhorn Royal Academy, Bangkok 10210, Thailand; 2 Radiation Oncology Department, Chulabhorn Hospital, Chulabhorn Royal Academy, Bangkok 10210, Thailand

In the original publication [1], the reference number 6 [2] was incorrectly cited. The citation has now been corrected in References and should read as follows:

6. Paulbeck, C.; Griffin, K.; Lee, C.; Cullings, H.; Egbert, S.D.; Funamoto, S.; Sato, T.; Endo, A.; Hertel, N.; Bolch, W.E. Dosimetric Impact of a New Computational Voxel Phantom Series for the Japanese Atomic Bomb Survivors: Pregnant Females. *Radiat. Res.* **2019**, *192*, 538–561.

With this correction, the order of references has not been changed. The authors state that the scientific conclusions are unaffected. This correction was approved by the Academic Editor. The original publication has also been updated.
